# Synergetic effects of DNA methylation and histone modification during mouse induced pluripotent stem cell generation

**DOI:** 10.1038/srep39527

**Published:** 2017-02-03

**Authors:** Guiying Wang, Rong Weng, Yuanyuan Lan, Xudong Guo, Qidong Liu, Xiaoqin Liu, Chenqi Lu, Jiuhong Kang

**Affiliations:** 1 Clinical and Translational Research Center of Shanghai First Maternity and Infant Health Hospital, Collaborative Innovation Center for Brain Science, Shanghai Key Laboratory of Signaling and Disease Research, School of Life Science and Technology, Tongji University, Shanghai, P.R. China; 2 Department of Biostatistics and Computational Biology, State Key Laboratory of Genetic Engineering, School of Life Sciences, Fudan University, Shanghai, P.R. China

## Abstract

DNA methylation and histone methylation (H3K27me3) have been reported as major barriers to induced pluripotent stem cell (iPSC) generation using four core transcription factors (Oct4, Sox2, Klf4, and c-Myc, termed OSKM). Here, to illustrate the possibility of deriving iPSCs via demethylation, as well as the exact effects of DNA methylation and histone modification on gene expression regulation, we performed RNA sequencing to characterize the transcriptomes of ES cells and iPSCs derived by demethylation with miR-29b or shDnmt3a, and carried out integrated analyses. Results showed that OSKM + miR-29b-iPSC was more close to ES cells than the others, and up-regulated genes typically presented with methylated CpG-dense promoters and H3K27me3-enriched regions. The differentially expressed genes caused by introduction of DNA demethylation during somatic cell reprogramming mainly focus on stem cell associated GO terms and KEGG signaling pathways, which may decrease the tumorigenesis risk of iPSCs. These findings indicated that DNA methylation and histone methylation have synergetic effects on regulating gene expression during iPSC generation, and demethylation by miR-29b is better than shDnmt3a for iPSC quality. Furthermore, integrated analyses are superior for exploration of slight differences as missed by individual analysis.

Induced pluripotent stem cells (iPSCs) can be generated from differentiated somatic cells by the ectopic expression of four core transcription factors (Oct4, Sox2, Klf4, and c-Myc, termed OSKM)^1^. Recent studies have raised serious concerns regarding the further application of iPSCs in personalized medicine, chief among which is the variation in the quality of iPSCs and their potential to differentiate into specific cellular lineages as compared to embryonic stem (ES) cells. Following the development of next-generation sequencing (NGS) technologies, the numbers of informative genomic, epigenomic, transcriptomic and proteomic datasets have increased rapidly^2^,^3^,^4^. Thus, screening various iPSCs derived using different factors and identifying key markers associated with pluripotency and differentiation potential have become prerequisites for the further application of iPSCs^5^. Furthermore, how to effectively utilize these omics data sets to illuminate the mechanisms of cell reprogramming is another research hotspot.

During cell reprogramming, erasing the genetic imprint of somatic cells is the first step in driving the cells to iPSCs. Epigenetic modifications such as DNA methylation play critical roles in the reestablishment of ES-specific gene expression pattern^6^. DNA methylation is mediated by DNA methyltransferases (Dnmts) such as Dnmt1 and Dnmt3a/3b, which are considered significant barriers to reprogramming. It has been reported that DNA demethylation is required for the reactivation of epithelial genes at the early stage of iPSC generation^6^. The non-specific inhibitor of Dnmts, 5-aza-2′-deoxycytidine, has facilitated the transition from somatic cells to pluripotent stem cells^7^,^8^,^9^. However, the expression levels of Dnmts are up-regulated during the late stage of iPSC generation, which is required to attain the developmental potential of fully pluripotent iPSCs^10^. Moreover, Dnmt3a or Dnmt3b conditional knockout ES cells fail to differentiate into three germ layers^11^. Recent studies have found that aberrant DNA hypermethylation could be responsible for silencing certain imprinted regions such as the *Dlk1-Dio3* locus, which is correlated with the developmental potentials of fully pluripotent iPSCs^12^,^13^.

Recently, increasing evidence has shown that microRNAs (miRNAs) are involved in the regulation of stem cell self-renewal and differentiation^14^,^15^. One of the most interesting miRNA family, miR-29b, plays a crucial role in human diseases by directly targeting Dnmt3a/3b^16^,^17^. The miR-29b/Sirt1 axis has been found to regulate the self-renewal of ES cells in response to reactive oxygen species (ROS)^18^. Our previous study elucidated the function and mechanism of miR-29b during iPSC generation, implicating it as a crucial mediator of Sox2 in the control of dynamic Dnmt3a/3b expression and in DNA methylation-related events, such as *Dlk1-Dio3* region transcription^19^. Furthermore, we found that the pluripotency and differentiation potentials of OSKM + miR-29b-iPSC are similar to that of ES cells. However, the differences between iPSCs generated with OSKM + miR-29b and OSKM + shDnmt3a remain to be clarified. Further systematic investigation of these iPSC lines may shed light on the effect of demethylation during somatic cell reprogramming.

During reprogramming, the somatic genome undergoes a variety of epigenetic modifications, including histone modification and DNA methylation^20^. It has been reported that histone modification generally precedes changes in DNA methylation^21^,^22^. The chromatin state of K4 (H3K4me3) and K27 (H3K27me3) effectively discriminates between activated and repressed genes, and has been used to distinguish somatic cells from pluripotent stem cells^23^. However, not all promoters associated with H3K4me3 are active, and often present as a bivalent state exhibiting both H3K4me3 and H3K27me3 modifications. The *de novo* methyltransferase Dnmt3a/3b are highly expressed in pluripotent stem cells, whereas H3K4me3 is enriched at promoters in iPSCs. Furthermore, H3K4me3 is known to be associated with DNA hypomethylation at many genomic loci in human iPSC and ES cells^24^. Thus, H3K4me3 and H3K27me3 are important epigenetic markers of successful reprogramming.

To explore the effects of DNA methylation, miRNAs, and histone modifications on the regulation of gene expression, we used RNA sequencing (RNA-seq) technology to obtain the whole transcriptomes of mouse ES cells and iPSCs derived using OSKM, OSKM + miR-29b, and OSKM + shDnmt3a. Our results showed that of OSKM + miR-29b-iPSC was more close to ES cells than the others. Further analyses showed that up-regulated genes typically presented with methylated CpG-dense promoters and H3K27me3-enriched regions, indicating the synergistic effects of DNA methylation and histone methylation on gene expression during iPSC generation.

## Results

### Raw data processing for RNA profiles of three iPSCs and ES cells

To clarify the exact differences and mechanisms underlying reprogramming with demethylation and the quality of pluripotency, we used iPSCs as previously reported^19^, derived using OSKM and in addition to demethylation with overexpressing miR-29b (OSKM + miR-29b) or repressing Dnmt3a using shRNA (OSKM + shDnmt3a). These three iPSC lines were referred to as iPSC (OSKM-iPSC), miR-29b (OSKM + miR-29b-iPSC), and shDnmt3a (OSKM + shDnmt3a-iPSC), respectively. Using high-throughput RNA-seq technology, we obtained the whole transcriptomes of four cell lines, including the three iPSC lines and ES cells (E14).

Through the workflow of RNA-seq analysis as described in the Methods section and Supplementary Figure S1, we obtained 27–33 M reads that uniquely mapped to the mouse genome (mm 10). The details of mapping were shown in Supplementary Table S1. According to Ensembl genome annotation (Mus_musculus.GRCm38.73.gtf), the raw transcriptomes contained 37,347 genes. After filtering low signal genes (Max _(FPKM)_ < 0.5), there were 17,638 genes remained.

### RNA profiles of iPSCs derived by demethylation are more close to ES cells

**Figure 1:**
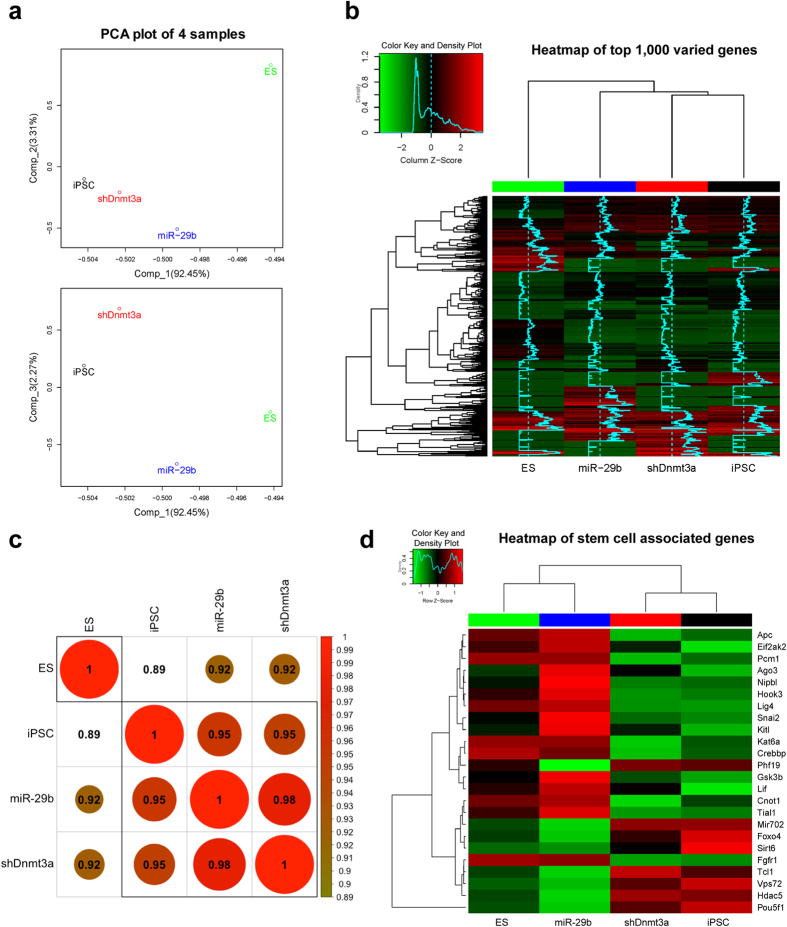
RNA profiles of iPSCs derived by demethylation are more close to ES cells. (**a**) A PCA plot explained according to the first principle component and second/third principle component via profiling all gene expression after filtering low signals. iPSC, OSKM-iPSC; miR-29b, OSKM + miR-29b-iPSC; shDnmt3a, OSKM + shDnmt3a-iPSC. (**b**) A heatmap generated using the top 1,000 differentially expressed genes among the four cell lines. Scaled by column. (**c**) The Pearson correlation coefficient clustering on the 238 genes associated with pluripotency, proliferation, and differentiation of stem cells. (**d**) The clustered heatmap of 24 critical genes in stem cells. Scaled by row.

To explore the similarity of the four transcriptomes, we performed the following analyses. Results of the principal component analysis (PCA) indicated that the expression profiles of the three iPSC lines are more similar (Fig. 1a). It was further confirmed by the clustered heatmap using UPGMA (average) on euclidean distance for the top 1,000 varied genes (according to standard deviation) among the four cell lines (Fig. 1b). Based on annotation of GO database, there are 238 genes associated with pluripotency, proliferation, and differentiation of stem cells observed in our RNA-seq data. Further analysis with Pearson correlation coefficient clustering on these 238 genes showed that OSKM + miR-29b-iPSC and OSKM + shDnmt3a-iPSC were more close to ES cells than OSKM-iPSC (Fig. 1c). Moreover, as shown in Fig. 1d, the clustered heatmap of 24 critical genes associated with stem cells indicated that OSKM + miR-29b-iPSC was more close to ES cells than the other iPSCs. These findings suggested that iPSCs derived with demethylation were more close to ES cells, and using miR-29b maybe better than shDnmt3a for demethylation during iPSC generation.

### Differentially expressed gene (DEG) analysis

**Figure 2:**
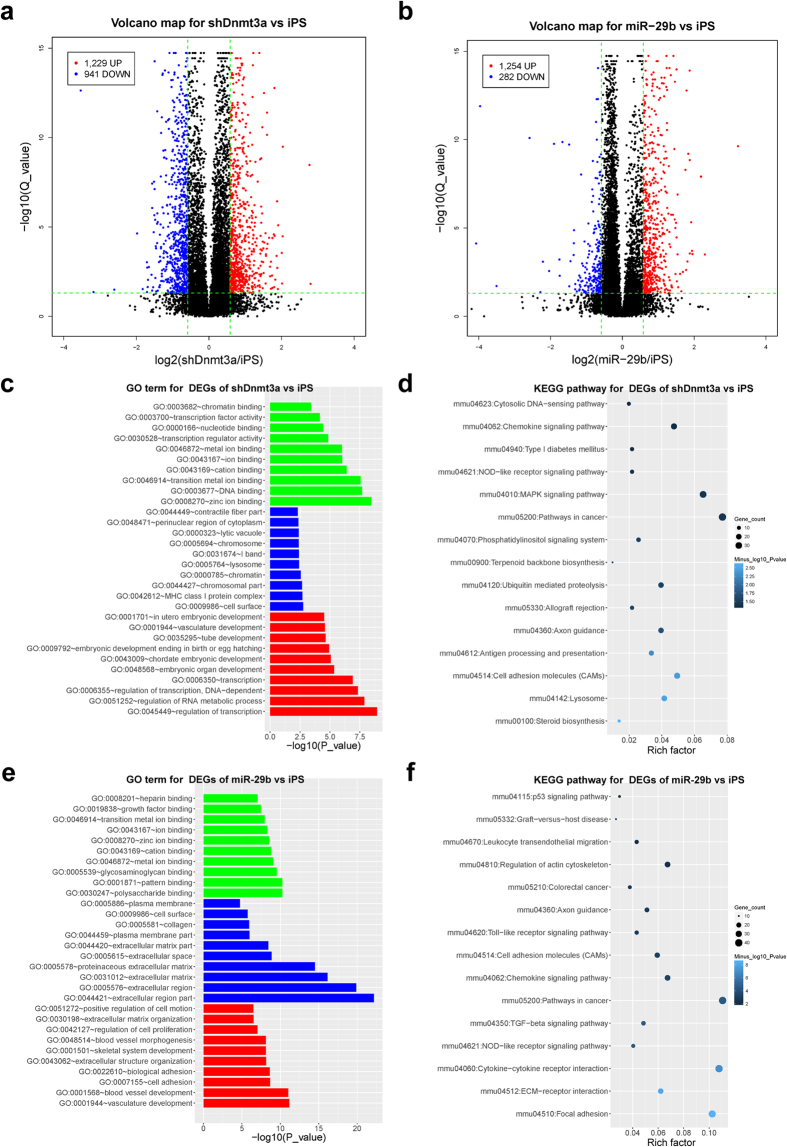
Demethylation during somatic cell reprogramming improves the quality of iPSCs. (**a**) A volcano plot of the DEGs between OSKM + shDnmt3a-iPSC and OSKM-iPSC; the x-axes index the log_2_(fold-change), and the y-axes index the –log(Q_value). (**b**) A volcano plot for DEGs between OSKM + miR-29b-iPSC and OSKM-iPSC. (**c**) GO analysis of DEG enrichment between OSKM + shDnmt3a-iPSC and OSKM-iPSC (colors: green for mf, blue for cc, red for bp). (**d**) KEGG pathway analysis of DEG enrichment between OSKM-shDnmt3a-iPSC and OSKM-iPSC. (**e**) GO analysis of DEG enrichment between OSKM + miR-29b-iPSC and OSKM-iPSC (colors: green for mf, blue for cc, red for bp). (**f**) KEGG pathway analysis of DEG enrichment between OSKM + miR-29b-iPSC and OSKM-iPSC. iPSC, OSKM-iPSC; miR-29b, OSKM + miR-29b-iPSC; shDnmt3a, OSKM + shDnmt3a-iPSC.

DEG analyses were performed using cufflink software (cuffdiff)^25^. Multiple tests were adjusted using false discovery rate (FDR) methods, and a cutoff of FDR < 0.05 was considered as statistically significant. When comparing the RNA profiles of OSKM + miR-29b-iPSC or OSKM + shDnmt3a-iPSC to that of OSKM-iPSC, a threshold of 1.5-fold was considered as biologically significant. As shown in the volcano maps, we obtained 1,229 genes up-regulated and 941 genes down-regulated in the group of OSKM + shDnmt3a-iPSC versus OSKM-iPSC (Fig. 2a), whereas 1,254 genes up-regulated and 282 genes down-regulated in OSKM + miR-29b-iPSC versus OSKM-iPSC (Fig. 2b).

### Demethylation during somatic cell reprogramming improves the quality of iPSCs

To identify the precise effects of demethylation during somatic cell reprogramming on iPSC quality, we performed further enrichment analyses. The results of GO enrichment for the DEGs of OSKM + shDnmt3a-iPSC versus OSKM-iPSC suggested that the DEGs induced by shDnmt3a were involved in regulation of transcription, development, and DNA binding (Fig. 2c and Supplementary Fig. S2a). Moreover, the KEGG pathway enrichment analyses indicated that these DEGs tended to focus on cell adhesion, MAPK signaling, chemokine signaling, and cancer pathways (Fig. 2d).

To investigate whether the GO term and KEGG pathway enrichment of DEGs in OSKM + miR-29b-iPSC has a similar pattern to that of DEGs in OSKM + shDnmt3a-iPSC, we performed further analysis on the DEGs caused by miR-29b. Results showed that the GO term and KEGG pathway of DEGs caused by miR-29b was similar to that of shDnmt3a. The GO analysis suggested that these DEGs were linked to development, cell adhesion, cell proliferation, and ion binding (Fig. 2e and Supplementary Fig. S2b). Moreover, KEGG pathway enrichment analyses showed that the DEGs tended to take part in stem cells associated key signaling pathways such as focal adhesion, cell adhesion, TGF-beta signaling, p53 signaling, and the MAPK signaling pathway (Fig. 2f). These findings indicated that introduction of demethylation via shDnmt3a or miR-29b during somatic cell reprogramming improved the quality of the derived iPSCs. Interestingly, DEGs caused by miR-29b mainly focus on stem cell related signaling pathways.

### Further analyses of DEGs between OSKM + shDnmt3a-iPSC and OSKM-iPSC

#### CpG methylation within the gene body is mainly affected by shDnmt3a

**Figure 3:**
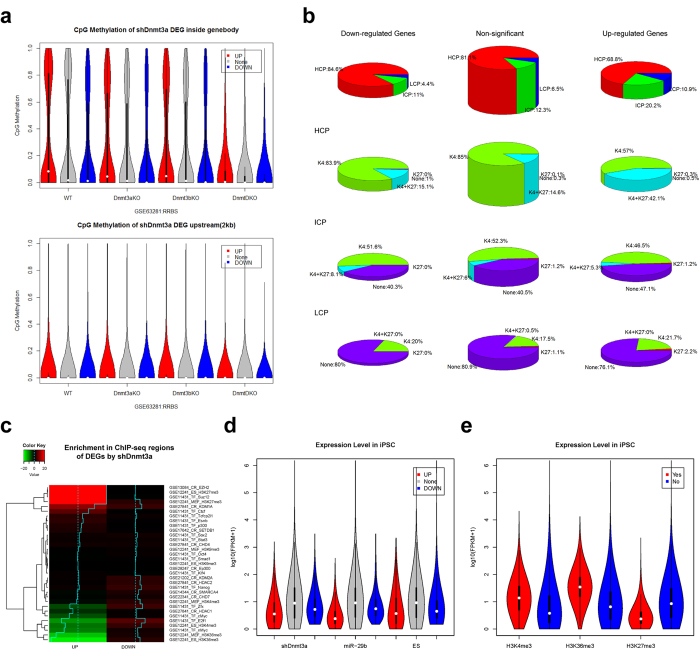
Further analysis for methylation status and promoter characteristics of DEGs between OSKM + miR-29b-iPSC and OSKM-iPSC. (**a**) A vioplot of the distribution of CpG methylation upstream and within the gene body for DEGs induced by shDnmt3a. WT, wild-type ES cells; Dnmt3aKO, knockout of Dnmt3a; Dnmt3bKO, knockout of Dnmt3b; DnmtDKO, knockout of Dnmt3a/3b. (**b**) A pie plot of the distribution of promoter characteristics (including the CpG density group and K4 and K27 signals) among DEGs induced by shDnmt3a. (**c**) A heatmap of DEG enrichment induced by shDnmt3a on ChIP-Seq regions. The Fisher test was used for statistical analysis: (log_2_(fold-change)) (−log_10_(P_value)). Red indicates overrepresentation, blue indicates underrepresentation, and color intensity indicates the P value of the Fisher test. (**d**) A vioplot of the expression level distribution in iPSCs among three DEGs. (**e**) A vioplot of the expression level distribution in iPSCs among genes with H3K4me3, H3K36me3 and H3K27me3 signals.

To explore whether the genes up-regulated by shDnmt3a were controlled by a high methylation level and demethylated by shDnmt3a, we utilized the reduced representation bisulfite sequencing (RRBS) data (GSE63281)^26^ generated from Dnmt3a knockout (Dnmt3aKO), Dnmt3b knockout (Dnmt3bKO), and double knockout of Dnmt3a and Dnmt3b (DnmtDKO) ES cells in the following analysis. Interestingly, the methylation level of CpG sites inside the gene body displayed a bimodal distribution in wild-type (WT) ES cells as either “largely unmethylated” or “largely methylated”. The up-regulated DEGs tended to contain more methylated genes than down-regulated DEGs, and the CpG methylation level decreased after Dnmt3a or Dnmt3b depletion, especially in Dnmt3a/3b double-knockout cells (Fig. 3a). Results also showed that the CpG methylation level upstream of these genes was low in ES cells and no difference was markedly observed between up-regulated DEGs and others (Fig. 3a). Furthermore, these upstream methylation signals had no efficient response to Dnmt3a/3b knockout. The methylation analysis for 238 stem cell associated genes was shown in Supplementary Figure S3a. These results suggested that only CpG methylation within the gene body was associated with the up-regulated DEGs of OSKM + shDnmt3a-iPSC versus OSKM-iPSC.

#### Characteristics of promoter regions in DEGs affected by shDnmt3a

During iPSC generation, knockdown of Dnmt3a could decrease the level of DNA methylation across the genome, especially at CpG sites in promoter regions. In mammals, the methylation level of CpG within promoter regions is closely and negatively associated with gene expression. To identify the CpG density at promoter regions of DEGs affected by shDnmt3a, we performed the following analysis. Based on the CpG density of promoter regions, Mikkelsen and colleagues classified gene promoters into three groups, i.e., those containing high, intermediate or low CpG density promoters (HCP, ICP, and LCP) (GSE8024)^23^. Regarding the details of binary methylation signals, CpG sites in HCP promoters tend to be unmethylated in ES cells, while those in LCP/ICP promoters tend to be methylated. As expected, these up-regulated genes in OSKM + shDnmt3a-iPSC (Fig. 3b) tended to have more LCP promoters (10.9%) and ICP promoters (20.2%) (Chi-square = 119.20, df = 4, p_value = 7.9 × 10^−25^), similar to those routinely methylated and silenced in ES cells.

Moreover, as shown in Fig. 3b, we found that there were a greater proportion of genes with K4-K27 among up-regulated genes with HCP (42.1%) than other HCP genes (such as 15.1% of down-regulated genes with HCP). These results suggested that global demethylation by shDnmt3a during iPSC generation tended to recover silenced genes with LCP or ICP promoters, while HCP was accompanied with K4-K27. In addition to DNA methylation of promoters, histone modifications of K27 were also found to be related to shDnmt3a.

Further, to identify the enrichment signals of transcriptional regulation for DEGs, we collected the ChIP-Seq data of chromatin regulators (CR database)^27^, transcription factors (GSE11431)^28^, and histone modifications (GSE12241)^23^ in ES and mouse embryonic fibroblast (MEF) cells. Results showed that the up-regulated genes by shDnmt3a tended to be suppressed genes in ES cells accompanied by H3K27me3 (Fig. 3c). Responsible for catalyzing H3K27me3, members of the polycomb repressive complex 2 (PRC2), Ezh2 and Suz12, were also highly associated with these up-regulated genes (Fig. 3c). In addition, up-regulated genes tended to have less active genes in ES cells with H3K4me3 and H3K36me3. KDM1A (LSD1) was also found to be enriched among the up-regulated genes, which catalyze H3K4me2 and inhibit H3K4me3. Additionally, the up-regulated genes included fewer targets of c-Myc/n-Myc and E2f1, as related to the quality of iPSCs.

It is clear that the statistical method based on fold-change to detect up-regulated DEGs tends to find more DEGs with low expression level in control samples. Unexpectedly, the down-regulated DEGs did not tend to show high expression level in OSKM-iPSC (Fig. 3d). Using histone modification data (GSE12241), we found that the active genes with K4-K36 signals tended to show high expression level, while the repressive genes with K27 signals tended to show low expression level (Fig. 3e).

To explore whether the relationship between up-regulated genes and K27 signals caused the low expression level observed in OSKM-iPSCs, we used a single logistic linear model, and found that genes with low expression level in OSKM-iPSC (logFPKM_iPSC) and H3K27me3 without H3K4me3 and H3K36me3 signals tended to be up-regulated by shDnmt3a (Table 1). Furthermore, based on multiple logistic regressions, we found that except H3K4me3 modification, the other three factors significantly contributed to the up-regulated genes (Table 1). Analysis on down-regulated genes suggested that genes with low expression level in OSKM-iPSC, H3K4me3, and H3K36me3 signals tended to be down-regulated. In addition, these three factors contributed independently to the down-regulated genes induced by shDnmt3a.

### Further analyses of DEGs between OSKM + miR-29b-iPSC and OSKM-iPSC

#### Characteristics of promoter regions in DEGs by miR-29b is similar but distinct to shDnmt3a

**Figure 4:**
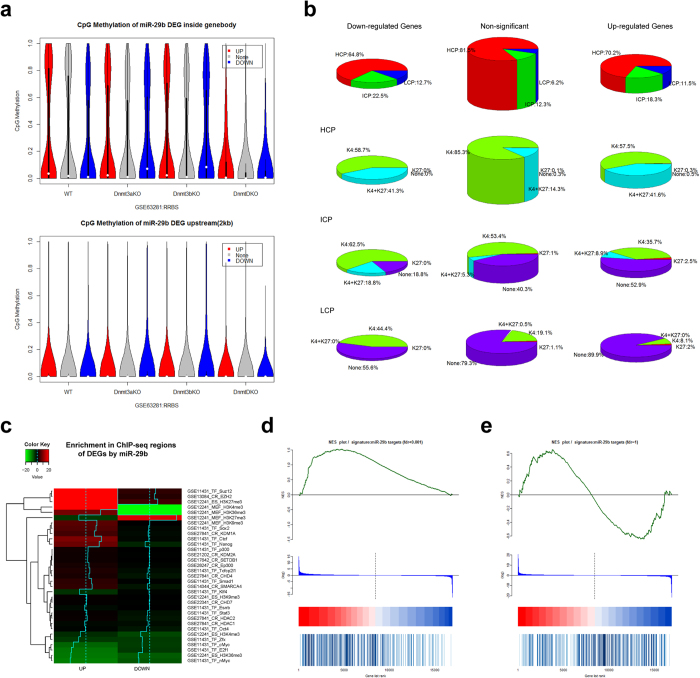
Further analyses for methylation status and promoter characteristics of DEGs between OSKM + miR-29b-iPSC and OSKM-iPSC. (**a**) A vioplot of the distribution of CpG methylation upstream and within the gene body among DEGs induced by miR-29b. WT, wild-type ES cells; Dnmt3aKO, knockout of Dnmt3a; Dnmt3bKO, knockout of Dnmt3b; DnmtDKO, double knockout of Dnmt3a/3b. (**b**) A pie plot of the distribution of promoter characteristics (including the CpG density group as well as K4 and K27 signals) among DEGs induced by miR-29b. (**c**) A heatmap of DEG enrichment by miR-29b on ChIP-Seq regions. (**d**) A GSEA plot of miR-29b targets (log_2_(fold-change)) between miR-29b and iPSCs. (**e**) A GSEA plot of miR-29b targets (log_2_(fold-change)) between shDnmt3a and iPSCs.

To investigate whether there is similar pattern of DEGs in group of OSKM + miR-29b-iPSC versus OSKM-iPSC and OSKM + shDnmt3a-iPSC versus OSKM-iPSC, we performed further analysis on DEGs caused by miR-29b. The results showed no significant DNA methylation pattern upstream or within the gene body of up-regulated genes (Fig. 4a and Supplementary Fig. S3b). In addition, the up-regulated genes tended to show ICP or LCP promoters and accompanied K27 signals (Fig. 4b). Different from OSKM + shDnmt3a-iPSC, down-regulated genes in OSKM + miR-29b-iPSC also tended to show more ICP (22.5%) or LCP (12.7%) promoters and K27 signals (Fig. 4b). Furthermore, the up-regulated genes in OSKM + miR-29b-iPSC tended to be associated with H3K27me3 in ES cells, and also enriched with binding of Ezh2 and Suz12 (Fig. 4c). The up-regulated genes were also associated with H3K4me3 and H3K36me3 signals in ES cells, as well as fewer targets of Myc and E2f1. Different from OSKM + shDnmt3a-iPSC, DEGs in OSKM + miR-29b-iPSC were associated with targets of pluripotent transcription factors Sox2, Ctcf and Nanog. Furthermore, except for H3K36me3 modification, the other three factors of logFPKM_iPSC, H3K4me3 and H3K27me3 were significantly and independently contributed to the up-regulated genes, based on multiple logistic regressions (Table 2).

#### Targets of miR-29b are mainly up-regulated DEGs by shDnmt3a

To investigate whether the target genes of miR-29b are directly regulated by miR-29b or indirectly affected by DNA demethylation, we collected 5,332 predicted targets and 153 validated targets of miR-29b from the miRwalk database^29^. Finally, there were 300 genes obtained by the combination of validated targets and predicted targets using more than seven methods. Interestingly, these miR-29b targets tend to be up-regulated in OSKM + miR-29b-iPSC (Fig. 4d). There was also a larger proportion of genes up-regulated in OSKM + shDnmt3a-iPSC (Fig. 4e), further confirming that these genes are mainly regulated by DNA methylation.

#### The specific DEGs regulated by shDnmt3a and miR-29b

**Figure 5:**
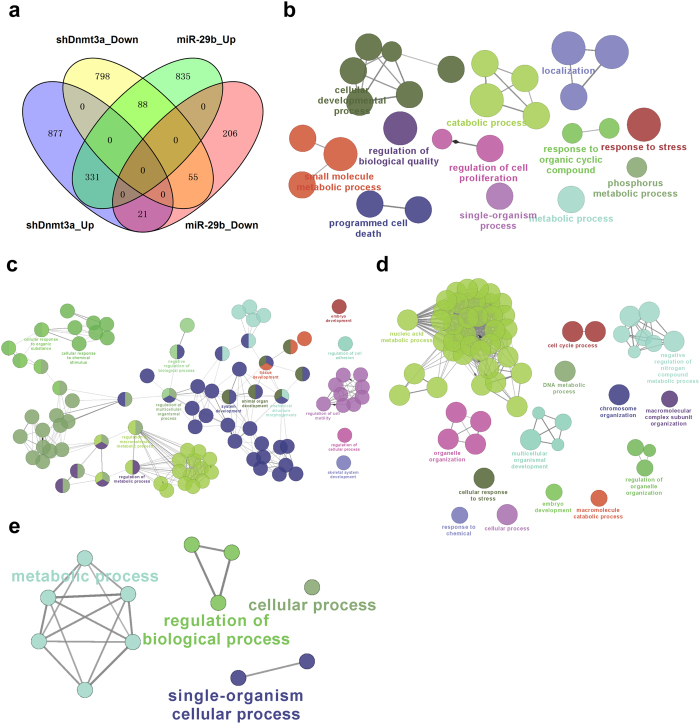
Further analyses of specific DEGs regulated by shDnmt3a and miR-29b. (**a**) A Venn diagram of the distribution of DEGs induced by both shDnmt3a and miR-29b. (**b**) A ClueGO plot (cytoscape app) in GO_BP for only genes up-regulated by shDnmt3a. (**c**) A ClueGO plot (cytoscape app) in GO_BP for only genes up-regulated by miR-29b. (**d**) A ClueGO plot (cytoscape app) in GO_BP for only genes down-regulated by shDnmt3a. (**e**) A ClueGO plot (cytoscape app) in GO_BP for only genes down-regulated by miR-29b. miR-29b, OSKM + miR-29b-iPSC; shDnmt3a, OSKM + shDnmt3a-iPSC.

As shown in Fig. 5a, the overlapping patterns of DEGs in OSKM + miR-29b-iPSC versus OSKM-iPSC and OSKM + shDnmt3a-iPSC versus OSKM-iPSC were presented in the Venn diagram. There were 331 up-regulated and 55 down-regulated genes that overlapped between the two iPSCs derived with the demethylation method. To identify the specific DEGs regulated by miR-29b or shDnmt3a, we carried out further analysis of the differences between OSKM + miR-29b-iPSC and OSKM + shDnmt3a-iPSC. Results showed that there were 877 genes and 835 genes that were specifically up-regulated in OSKM + shDnmt3a-iPSC versus OSKM-iPSC and OSKM + miR-29b-iPSC versus OSKM-iPSC, while 798 genes and 206 genes were down-regulated only in OSKM + shDnmt3a-iPSC versus OSKM-iPSC and OSKM + miR-29b-iPSC versus OSKM-iPSC, respectively.

Further GO and KEGG pathway analyses indicated that the specifically up-regulated genes in OSKM + shDnmt3a-iPSC were enriched in catabolic process, metabolic process, cellular developmental process, single-organism transport, cell proliferation and cell death (Fig. 5b). However, the specifically up-regulated genes in OSKM + miR-29b-iPSC were mainly enriched in development, metabolic process, cell adhesion and motility (Fig. 5c). Moreover, the specifically down-regulated genes in OSKM + shDnmt3a-iPSC were enriched in macromolecular metabolic process, DNA metabolic processes, nucleic acid metabolic process and organelle organization (Fig. 5d), while the down-regulated genes in OSKM + miR-29b-iPSC were enriched in the regulation of metabolic, biological and cellular processes (Fig. 5e). These findings suggested that miR-29b and shDnmt3a played similar but distinct roles in regulating gene expression and iPSC generation.

## Discussion

During iPSC generation, changes in epigenetic modifications such as DNA methylation are crucial for the reestablishment of ES-specific gene expression pattern^7^. Here, to systematically characterize the quality of iPSCs derived using demethylation via overexpression of miR-29b or knockdown of Dnmt3a, as well as the exact effects of DNA methylation and histone modification on regulating gene expression, we performed RNA-seq across the whole transcriptomes of ES cells and iPSCs derived by demethylation. Results showed that the synergetic role of DNA methylation and histone modification is crucial for controlling gene expression and that demethylation treatments tend to activate genes with methylated and H3K27me3-enriched promoters in ES cells. Thus, demethylation may be the first condition required to activate gene expression during iPSC generation.

During iPSC generation, erasing the genomic imprint of somatic cells is the first step in inducing iPSCs. DNA demethylation is required for the reactivation of epithelial genes at the early stage of iPSC generation, while activation of the imprinted regions of the *Dlk1-Dio3* locus is correlated with the developmental potential of fully pluripotent iPSCs^6^,^12^,^13^. DNA methyltransferases are considered significant barriers for reprogramming, but required for the developmental potential of fully pluripotent iPSCs^10^. Therefore, introduction of demethylation via Dnmt3a knockout will therefore lead to unexpected side effects, whereas knockdown of Dnmt3a by miRNA and shRNA will not completely deplete methylation and maybe better for iPSC generation. Recently, miR-29b has been found to regulate the self-renewal of ES cells during ROS treatment and was found to be critically involved in DNA methylation-related reprogramming events^18^,^19^. As previously reported^19^, we confirmed that three iPSC lines with similar pluripotency and differentiation potentials exhibit similar gene expression profiles that are distinct from that of ES cells. Furthermore, the up-regulated genes contain fewer tumor-related transcription factors such as cMyc/nMyc and E2f1, indicating that demethylation by miRNA and shRNA is better for the quality of iPSCs. GO and KEGG pathway analyses also revealed that demethylation is better for iPSC generation and for decreasing their tumorigenic potential.

It has been reported that CpG sites in HCP promoters tend to be unmethylated, while CpG sites in LCP/ICP promoters tend to be methylated^23^. As expected, we found that the up-regulated genes tended to contain more LCP/ICP promoters, which were typically silenced and methylated. It has been reported that DNA methylation is linked with histone modification^20^, and the chromatin state of K4 (H3K4me3) and K27 (H3K27me3) effectively discriminates genes as activated or repressed^23^. Further analyses showed that K27 modifications were related to shDnmt3a, while up-regulated genes usually harbored H3K27me3 promoters. Global demethylation by shDnmt3a and miR-29b tended to recover silenced genes with LCP or ICP promoters, while HCP promoters were accompanied by K4-K27. A specific modification pattern termed a “bivalent domain”, consisting of a large region of H3K27 methylation harboring smaller regions of H3K4 methylation, keeps genes poised for activation and has been identified in ES cells^30^, highlighting the importance of the DNA sequence in defining the initial epigenetic landscape and suggesting a novel chromatin-based mechanism for maintaining pluripotency. By combining various online data sets, we confirmed that the DEGs up-regulated by shDnmt3a and miR-29b tend to be poised for activation, while the synergy of DNA methylation and histone modification is critical for controlling gene expression.

Following the development of NGS technologies, high-throughput sequencing has become an increasingly important tool for biological research. Faced with a rapidly increasing amount of genomic, epigenomic, transcriptomic and proteomic data, effectively integrating these omics data sets to best illuminate the mechanisms of development, cell reprogramming and cell fate determination has become a major concern. In this study, various levels of high-throughput data, including RRBS on DNA methylation, ChIP-seq on histone modification, and RNA-seq on transcriptomes, were used to perform systematic and comprehensive analyses in combination with multiple data analysis methods. Integrating various levels of data and multiple analysis methods is advantageous to explore slight differences, associations and mechanisms that a single method cannot identify. We identified not only the synergetic role of DNA methylation and histone modification in regulating gene expression, but also that demethylated treatments during iPSC generation prefer to activate genes with methylated and H3K27me3-enriched promoters. Thus, the combination of high-throughput sequencing, basic experiments and bioinformatics analyses may help to solve the remaining questions associated with iPSCs.

## Methods

### Cell lines and cell culture

Mouse iPSCs including OSKM-iPSC, OSKM + miR-29b-iPSC, and OSKM + shDnmt3a-iPSC were previously reported and obtained using the traditional transcriptional factors OSKM, overexpressing miR-29b with OSKM, and shDnmt3a with OSKM, respectively^19^. Retroviral plasmids were used for overexpression of miR-29b and knockdown of Dnmt3a. Mouse E14 cells^31^ were purchased from Cell Bank Type Culture Collection of Chinese Academy of Sciences (CBTCCCAS, Shanghai, China). All iPSCs and ES cells were maintained in knockout serum replacement (KOSR) medium consisting of knockout-DMEM (Gibco) containing 20% KOSR (Gibco), 1× P/S, 1× nonessential amino acids (NEAA) (Thermo), 1× L-glutamine (Thermo) and β-mercaptoethanol (Gibco) with leukemia inhibitory factor (LIF) (Millipore) on 0.1% gelatin (Sigma)-coated plates. The iPSCs were maintained on feeder layers of mitomycin C (Sigma)-treated MEF cells and were passaged every two days. All methods were carried out in accordance with the relevant guidelines, and all experiments were approved by the Institutional Animal Care and Use Committee of Tongji University.

### RNA-seq library generation

The RNA-seq library was generated according to the methods described in a study by Head *et al*.^32^.

### RNA-seq workflow

As shown in Supplementary Figure S1, before aligning RNA-seq reads to the mouse genome, poor quality bases were trimmed using sickle.pe (pair-end) (v1.29, https://github.com/najoshi/sickle) with the parameters (−q 20, −l 30). Approximately two of every three raw reads were maintained after the trimming step (Supplementary Table S1). Then, trimmed reads were mapped to the mouse reference genome (mm 10) using Tophat (2.0.7) with the default parameters and Ensemble genome annotation (Mus_musculus.GRCm38.73.gtf)^33^. For mapping quality control, RSeQC (v2.6) was used^34^. The trimming step allowed us to supply 2.5 times the unique mapped reads as is possible without trimming (Supplementary Table S1). Next, the expression level (FPKM, Fragments Per Kilobase of exon per Million fragments mapped) of each gene was estimated using Cufflinks (v2.0.2) software^25^. DEGs were detected using Cuffdiff 35. Multiple tests were adjusted using FDR methods, and a cutoff FDR <0.05 was chosen for statistical significance. Finally, the Bioconductor ‘cummeRbund’ package (v 2.10.0) was applied to manage, visualize and integrate all of the results produced by Cuffdiff.

### High-throughput datasets

Three datasets describing methylation, promoter characteristics and ChIP-Seq of regulatory regions in ES cells were obtained from the GEO database. First, the DNA methylation dataset (GSE63281) used RRBS to detect the methylation profile of ES cells, including wild-type cells and Dnmt1, Dnmt3a, Dnmt3b, double- and triple-knockouts^26^. Second, Mikkelsen and colleagues classified gene promoters according to CpG density into three groups: high, intermediate and low CpG density promoters (HCP, ICP and LCP) (GSE8024)^23^. It has been found that the chromatin state of K4 (H3K4me3) and K27 (H3K27me3) effectively discriminates genes as activated or repressed^23^. Virtually all HCPs (99%) were associated with intervals of significant H3K4me3 enrichment in ES cells. However, not all promoters associated with H3K4me3 were active. Among these promoters, 22% of the HCPs were bivalent, exhibiting both H3K4me3 and H3K27me3, a repressive signal. Finally, ChIP-Seq datasets of chromatin regulators (CR database http://compbio.tongji.edu.cn/cr/)^27^, transcription factors (GSE11431)^28^, and histone modifications (GSE12241)^23^ in ES/MEF cells were downloaded to identify enrichment signals among DEGs.

### Statistical analyses and plots

All statistical analyses and graphs were completed using R packages. The R packages including princomp, Heatplus, venn, corrplot and vioplot were used to generate PCA, heatmap, Venn, correlation and violin plots, respectively. The Fisher’s exact test was used for enrichment in ChIP-Seq, and the logistic regression model was completed using an R package. GSEA graphs were plotted with the R package phenoTest for gene enrichment analysis^36^.

### GO and KEGG pathway enrichment analyses

GO and KEGG pathway enrichment analyses of the relevant gene sets were completed using DAVID web servers^37^ and the Cytoscape app^38^. Results of GO terms were illustrated as bar plots, while the results of KEGG enrichment were presented as rich factor plots by ggplot2 package in R. The ClueGO was used to visualize the non-redundant biological terms in functionally grouped network, as highly clustered to reduce redundancy^39^.

## Additional Information

**How to cite this article:** Wang, G. *et al*. Synergetic effects of DNA methylation and histone modification during mouse induced pluripotent stem cell generation. *Sci. Rep.* **7**, 39527; doi: 10.1038/srep39527 (2017).

**Publisher's note:** Springer Nature remains neutral with regard to jurisdictional claims in published maps and institutional affiliations.

## Supplementary Material

Supplementary Information

## Figures and Tables

**Table 1:** Results of single and multiple logistic regression analyses of DEGs in OSKM + shDnmt3a-iPSC based on logFPKM_iPSC, K36, K4, and K27 signals.

	Crude OR (95%CI)	Adj. OR (95%CI)	P (Wald’s test)	P (LR-test)
DEGs up-regulated by shDnmt3a				
logFPKM_iPSC (cont. var.)	0.44 (0.39, 0.48)	0.51 (0.45, 0.56)	<0.001	<0.001
GSE12241_ES_H3K36me3: 1 vs 0	0.30 (0.22, 0.41)	0.52 (0.37, 0.71)	<0.001	<0.001
GSE12241_ES_H3K4me3: 1 vs 0	0.70 (0.62, 0.78)	0.90 (0.80, 1.03)	0.119	0.119
GSE12241_ES_H3K27me3: 1 vs 0	3.13 (2.57, 3.80)	2.31 (1.89, 2.83)	<0.001	<0.001
DEGs down-regulated by shDnmt3a				
logFPKM_iPSC (cont. var.)	0.70 (0.63, 0.77)	0.57 (0.51, 0.64)	<0.001	<0.001
GSE12241_ES_H3K36me3: 1 vs 0	1.43 (1.18, 1.73)	1.70 (1.37, 2.10)	<0.001	<0.001
GSE12241_ES_H3K4me3: 1 vs 0	1.31 (1.15, 1.49)	1.50 (1.30, 1.73)	<0.001	<0.001
GSE12241_ES_H3K27me3: 1 vs 0	1.02 (0.75, 1.41)	0.77 (0.55, 1.06)	0.109	0.098

**Table 2:** Results of single and multiple logistic regression analyses of DEGs in OSKM + miR-29b-iPSC based on logFPKM_iPSC, K36, K4, and K27 signals.

	Crude OR (95%CI)	Adj. OR (95%CI)	P (Wald’s test)	P (LR-test)
DEGs up-regulated by miR-29b				
logFPKM_iPSC (cont. var.)	0.19 (0.17, 0.21)	0.16 (0.13, 0.18)	<0.001	<0.001
GSE12241_ES_H3K36me3: 1 vs 0	0.48 (0.37, 0.62)	1.34 (1.02, 1.76)	0.036	0.042
GSE12241_ES_H3K4me3: 1 vs 0	0.81 (0.72, 0.91)	1.53 (1.34, 1.75)	<0.001	<0.001
GSE12241_ES_H3K27me3: 1 vs 0	3.14 (2.59, 3.81)	1.60 (1.31, 1.97)	<0.001	<0.001
DEGs down-regulated by miR-29b				
logFPKM_iPSC (cont. var.)	0.77 (0.65, 0.92)	0.98 (0.82, 1.16)	0.783	0.783
GSE12241_ES_H3K36me3: 1 vs 0	0.16 (0.07, 0.39)	0.22 (0.09, 0.54)	<0.001	<0.001
GSE12241_ES_H3K4me3: 1 vs 0	0.56 (0.44, 0.72)	0.61 (0.47, 0.79)	<0.001	<0.001
GSE12241_ES_H3K27me3: 1 vs 0	2.67 (1.82, 3.93)	2.74 (1.84, 4.10)	<0.001	<0.001

## References

[b1] Takahashi K. & Yamanaka S. Induction of pluripotent stem cells from mouse embryonic and adult fibroblast cultures by defined factors. Cell 126, 663–676 (2006). 16904174 10.1016/j.cell.2006.07.024

[b2] Doi A. . Differential methylation of tissue- and cancer-specific CpG island shores distinguishes human induced pluripotent stem cells, embryonic stem cells and fibroblasts. Nat. Genet. 41, 1350–1353 (2009). 19881528 10.1038/ng.471 PMC2958040

[b3] Guenther M. G. . Chromatin structure and gene expression programs of human embryonic and induced pluripotent stem cells. Cell Stem Cell 7, 249–257 (2010). 20682450 10.1016/j.stem.2010.06.015 PMC3010384

[b4] Kim K. . Epigenetic memory in induced pluripotent stem cells. Nature 467, 285–290 (2010). 20644535 10.1038/nature09342 PMC3150836

[b5] Chang G. . High-throughput sequencing reveals the disruption of methylation of imprinted gene in induced pluripotent stem cells. Cell Res. 24, 293–306 (2014). 24381111 10.1038/cr.2013.173 PMC3945885

[b6] Li R. . A mesenchymal-to-epithelial transition initiates and is required for the nuclear reprogramming of mouse fibroblasts. Cell Stem Cell 7, 51–63 (2010). 20621050 10.1016/j.stem.2010.04.014

[b7] Mikkelsen T. S. . Dissecting direct reprogramming through integrative genomic analysis. Nature 454, 49–55 (2008). 18509334 10.1038/nature07056 PMC2754827

[b8] Huangfu D. . Induction of pluripotent stem cells by defined factors is greatly improved by small-molecule compounds. Nat. Biotechnol. 26, 795–797 (2008). 18568017 10.1038/nbt1418 PMC6334647

[b9] Shi Y. . Induction of pluripotent stem cells from mouse embryonic fibroblasts by Oct4 and Klf4 with small-molecule compounds. Cell Stem Cell 3, 568–574 (2008). 18983970 10.1016/j.stem.2008.10.004

[b10] Pawlak M. & Jaenisch R. De novo DNA methylation by Dnmt3a and Dnmt3b is dispensable for nuclear reprogramming of somatic cells to a pluripotent state. Genes Dev. 25, 1035–1040 (2011). 21576263 10.1101/gad.2039011 PMC3093119

[b11] Jackson M. . Severe global DNA hypomethylation blocks differentiation and induces histone hyperacetylation in embryonic stem cells. Mol. Cell Biol. 24, 8862–8871 (2004). 15456861 10.1128/MCB.24.20.8862-8871.2004 PMC517875

[b12] Li W. . iPS cells generated without c-Myc have active Dlk1-Dio3 region and are capable of producing full-term mice through tetraploid complementation. Cell Res. 21, 550–553 (2011). 21321610 10.1038/cr.2011.25 PMC3193427

[b13] Liu L. . Activation of the imprinted Dlk1-Dio3 region correlates with pluripotency levels of mouse stem cells. J. Biol. Chem. 285, 19483–19490 (2010). 20382743 10.1074/jbc.M110.131995 PMC2885227

[b14] Lichner Z. . The miR-290-295 cluster promotes pluripotency maintenance by regulating cell cycle phase distribution in mouse embryonic stem cells. Differentiation 81, 11–24 (2011). 20864249 10.1016/j.diff.2010.08.002

[b15] Mathieu J. & Ruohola-Baker H. Regulation of stem cell populations by microRNAs. Adv. Exp. Med. Biol. 786, 329–351 (2013). 23696365 10.1007/978-94-007-6621-1_18 PMC3901537

[b16] Fabbri M. . MicroRNA-29 family reverts aberrant methylation in lung cancer by targeting DNA methyltransferases 3A and 3B. Proc. Natl. Acad. Sci. USA 104, 15805–15810 (2007). 17890317 10.1073/pnas.0707628104 PMC2000384

[b17] Schmitt M. J., Margue C., Behrmann I. & Kreis S. MiRNA-29: a microRNA family with tumor-suppressing and immune-modulating properties. Curr. Mol. Med. 13, 572–585 (2013). 22934851 10.2174/1566524011313040009

[b18] Xu Z. . The miR-29b-Sirt1 axis regulates self-renewal of mouse embryonic stem cells in response to reactive oxygen species. Cell Signal. 26, 1500–1505 (2014). 24657470 10.1016/j.cellsig.2014.03.010

[b19] Guo X. D. . microRNA-29b is a novel mediator of Sox2 function in the regulation of somatic cell reprogramming. Cell Res. 23, 142–156 (2013). 23266889 10.1038/cr.2012.180 PMC3541656

[b20] Cedar H. & Bergman Y. Linking DNA methylation and histone modification: patterns and paradigms. Nat. Rev. Genet. 10, 295–304 (2009). 19308066 10.1038/nrg2540

[b21] Mattout A., Biran A. & Meshorer E. Global epigenetic changes during somatic cell reprogramming to iPS cells. J. Mol. Cell Biol. 3, 341–350 (2011). 22044880 10.1093/jmcb/mjr028

[b22] Polo J. M. . A molecular roadmap of reprogramming somatic cells into iPS cells. Cell 151, 1617–1632 (2012). 23260147 10.1016/j.cell.2012.11.039 PMC3608203

[b23] Mikkelsen T. S. . Genome-wide maps of chromatin state in pluripotent and lineage-committed cells. Nature 448, 553–560 (2007). 17603471 10.1038/nature06008 PMC2921165

[b24] Nishino K. . Defining hypo-methylated regions of stem cell-specific promoters in human iPS cells derived from extraembryonic amnions and lung fibroblasts. PLoS One 5, e13017 (2010). 20885964 10.1371/journal.pone.0013017 PMC2946409

[b25] Trapnell C. . Differential gene and transcript expression analysis of RNA-seq experiments with TopHat and Cufflinks. Nat. Protoc. 7, 562–578 (2012). 22383036 10.1038/nprot.2012.016 PMC3334321

[b26] Liao J. . Targeted disruption of DNMT1, DNMT3A and DNMT3B in human embryonic stem cells. Nat. Genet. 47, 469–478 (2015). 25822089 10.1038/ng.3258 PMC4414868

[b27] Wang Q. . CR Cistrome: a ChIP-Seq database for chromatin regulators and histone modification linkages in human and mouse. Nucleic Acids Res. 42, D450–458 (2014). 24253304 10.1093/nar/gkt1151 PMC3965064

[b28] Chen X. . Integration of external signaling pathways with the core transcriptional network in embryonic stem cells. Cell 133, 1106–1117 (2008). 18555785 10.1016/j.cell.2008.04.043

[b29] Dweep H., Gretz N. & Sticht C. miRWalk database for miRNA-target interactions. Methods Mol. Biol. 1182, 289–305 (2014). 25055920 10.1007/978-1-4939-1062-5_25

[b30] Bernstein B. E. . A bivalent chromatin structure marks key developmental genes in embryonic stem cells. Cell 125, 315–326 (2006). 16630819 10.1016/j.cell.2006.02.041

[b31] Kuhn R., Rajewsky K. & Muller W. Generation and analysis of interleukin-4 deficient mice. Science 254, 707–710 (1991). 1948049 10.1126/science.1948049

[b32] Head S. R. . Library construction for next-generation sequencing: overviews and challenges. Biotechniques 56, 61–64, 66, 68, passim (2014). 24502796 10.2144/000114133 PMC4351865

[b33] Trapnell C., Pachter L. & Salzberg S. L. TopHat: discovering splice junctions with RNA-Seq. Bioinformatics 25, 1105–1111 (2009). 19289445 10.1093/bioinformatics/btp120 PMC2672628

[b34] Wang L., Wang S. & Li W. RSeQC: quality control of RNA-seq experiments. Bioinformatics 28, 2184–2185 (2012). 22743226 10.1093/bioinformatics/bts356

[b35] Trapnell C. . Transcript assembly and quantification by RNA-Seq reveals unannotated transcripts and isoform switching during cell differentiation. Nat. Biotechnol. 28, 511–515 (2010). 20436464 10.1038/nbt.1621 PMC3146043

[b36] Subramanian A. . Gene set enrichment analysis: a knowledge-based approach for interpreting genome-wide expression profiles. Proc. Natl. Acad. Sci. USA 102, 15545–15550 (2005). 16199517 10.1073/pnas.0506580102 PMC1239896

[b37] Huang D. W. . DAVID Bioinformatics Resources: expanded annotation database and novel algorithms to better extract biology from large gene lists. Nucleic Acids Res. 35, W169–175 (2007). 17576678 10.1093/nar/gkm415 PMC1933169

[b38] Lotia S., Montojo J., Dong Y., Bader G. D. & Pico A. R. Cytoscape app store. Bioinformatics 29, 1350–1351 (2013). 23595664 10.1093/bioinformatics/btt138 PMC3654709

[b39] Bindea G. . ClueGO: a Cytoscape plug-in to decipher functionally grouped gene ontology and pathway annotation networks. Bioinformatics 25, 1091–1093 (2009). 19237447 10.1093/bioinformatics/btp101 PMC2666812

