# Supplementary material for: Synergetic effects of DNA methylation and histone modification during mouse induced pluripotent stem cell generation
Source: Sci Rep. 2017 Feb 3;7:39527. doi: 10.1038/srep39527 (PMC5290738; doi:10.1038/srep39527)
Supplement: Supplementary Information [file srep39527-s1.pdf]

Supplementary Information

Synergetic effects of DNA methylation and histone modification during mouse induced pluripotent stem cell generation

Guiying Wang[1], Rong Weng[1], Yuanyuan Lan[1], Xudong Guo[1], Qidong Liu[1], Xiaoqin Liu[1], Chenqi Lu[2,*], Jiuhong Kang[1,*]

[1] Clinical and Translational Research Center of Shanghai First Maternity and Infant Health Hospital, Collaborative Innovation Center for Brain Science, Shanghai Key Laboratory of Signaling and Disease Research, School of Life Science and Technology, Tongji University, Shanghai, PR China.

[2] Department of Biostatistics and Computational Biology, State Key Laboratory of Genetic Engineering, School of Life Sciences, Fudan University, Shanghai, PR China.

*Corresponding authors: Chenqi Lu (email: luchenqi@fudan.edu.cn) or Jiuhong Kang (email: jhkang@tongji.edu.cn).

The supplementary information included Figure S1-S3 and Table S1.

Supplementary Figure S1. The analysis workflow for RNA sequencing data.

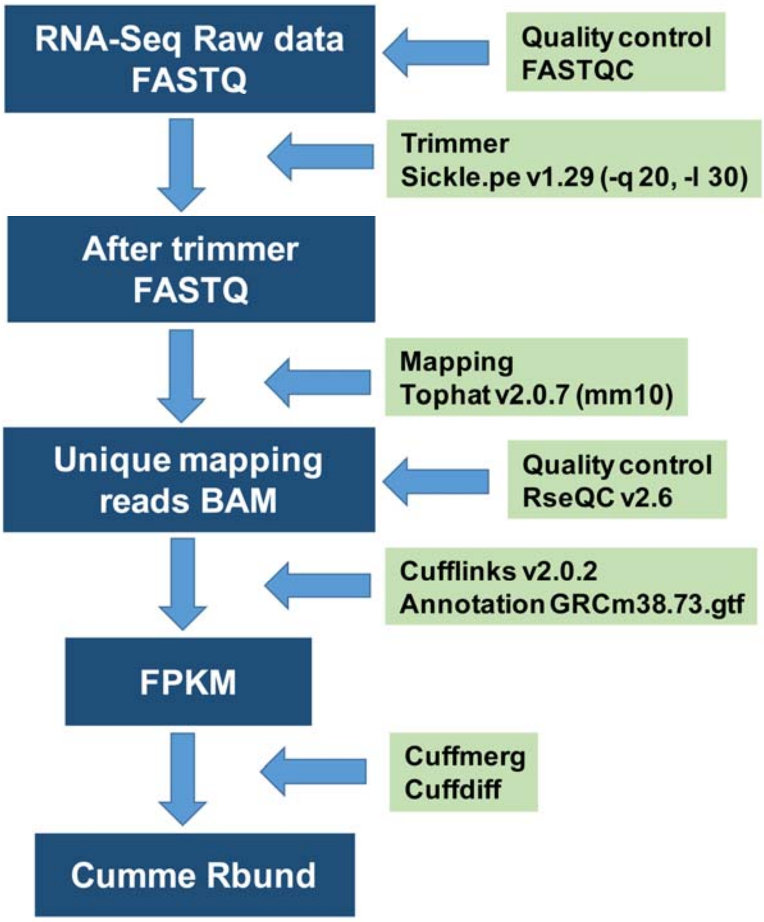

Supplementary Figure S2. Detailed development process among enriched GO terms of DEGs affected by demethylation.

(a) Development associated GO terms enrichment of DEGs between OSKM+shDnmt3a-iPSC and OSKM-iPSC.

(b) Development associated GO terms enrichment of DEGs between OSKM+miR-29b-iPSC and OSKM-iPSC.

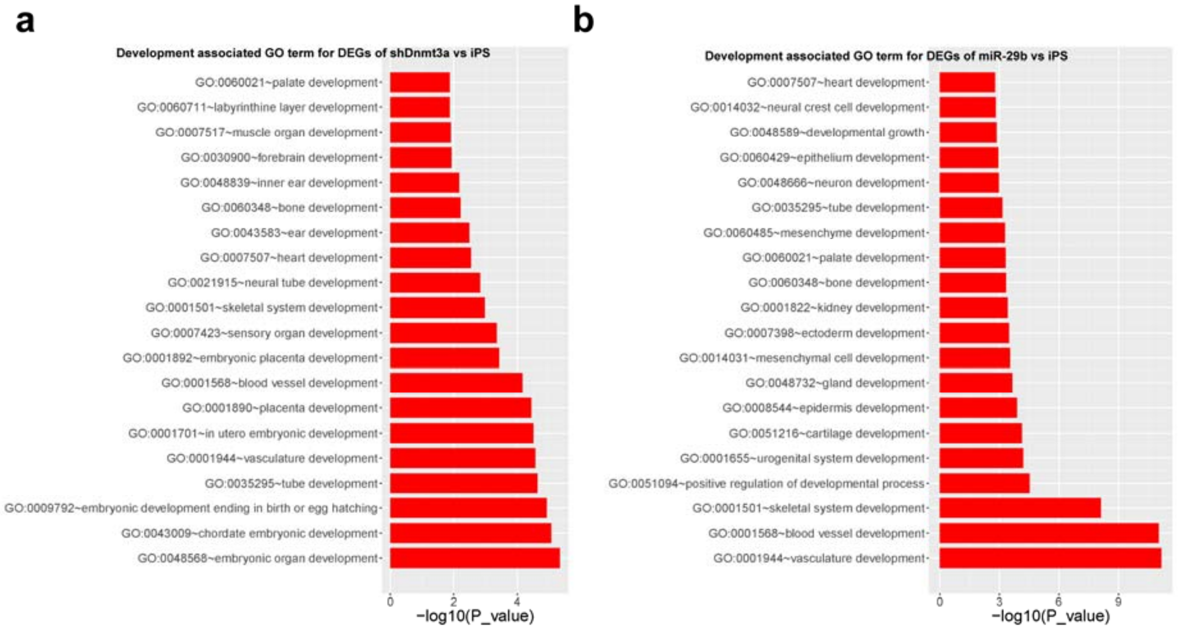

Supplementary Figure S3. Analysis for the methylation status of 238 stem cells associated genes with by voiplot.

(a) A vioplot of the distribution of CpG methylation upstream and within the gene body for 238 stem cells associated genes affected by shDnmt3a.

(b) A vioplot of the distribution of CpG methylation upstream and within the gene body for 238 stem cells associated genes affected by miR-29b. WT, wild-type ES cells; Dnmt3aKO, knockout of Dnmt3a; Dnmt3bKO, knockout of Dnmt3b; DnmtDKO, knockout of Dnmt3a/3b.

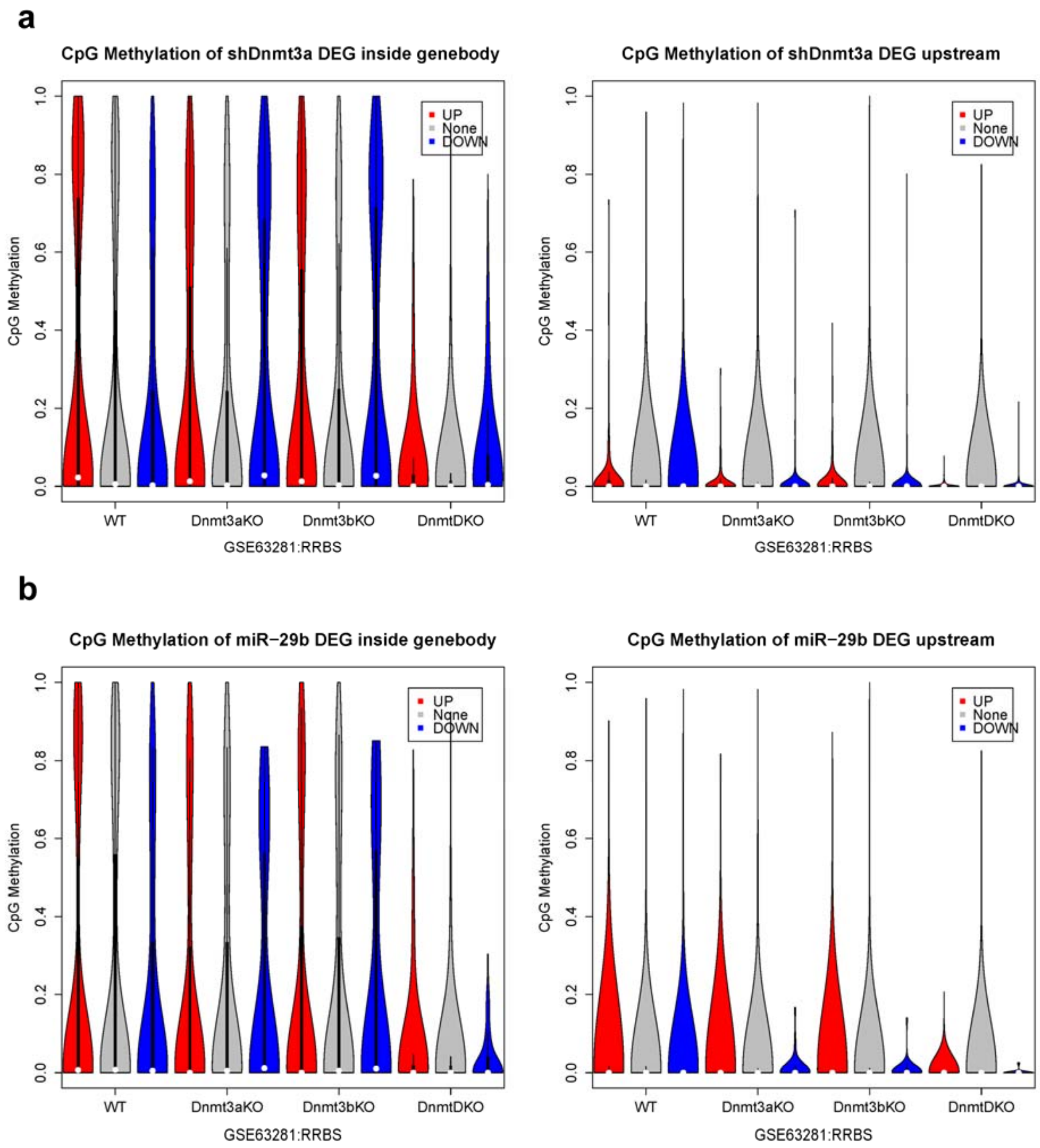
a
CpG Methylation of shDnmt3a DEG inside genebody
CpG Methylation of shDnmt3a DEG upstream
b
CpG Methylation of miR−29b DEG inside genebody
CpG Methylation of miR−29b DEG upstream
CpG Methylation
UP
None
DOWN
WT
Dnmt3aKO
Dnmt3bKO
DnmtDKO
GSE63281:RRBS

Supplementary Table S1. The summary of RNA-seq mapping results before and after trimming.

	Items		iPSCs		
		ES	OSKM	OSKM+miR-29b	OSKM+shDnmt3a
Raw data	NO. of paired reads	29600950	33203284	33547683	31659928
	Length	100	100	100	100
	GC%	53	52	51/52	53
Tophat	Unique mapped reads	10644037	12993441	13870191	11554076
	Mapped in pairs	2630890	3323642	3715850	2986708
After trimming	NO. of paired reads	16485482	18893004	19565387	17101260
	Length	30-100	30-100	30-100	30-100
	GC%	50	49	49	50
	No. of single reads	6429415	7081491	7067005	6739169
	Length	30-100	30-100	30-100	30-100
	GC%	51	51	50	51
	Total NO. of reads	39400379	44867499	46197779	40941689
	Usable %	66.55%	67.56%	68.85%	64.66%
Tophat	Unique mapped reads	27048541	32035451	33400756	29026534
	Mapped in pairs	8863714	10427406	11573606	10181962
	Ratio of Unique mapped reads	2.54	2.47	2.41	2.51
	Ratio of mapped in pairs	3.37	3.14	3.11	3.41
